# Rhegmatogenous retinal detachment in a patient with choroidal melanoma simulating choroidal detachment: a case report

**DOI:** 10.1186/s13256-018-1921-7

**Published:** 2018-12-25

**Authors:** Satoru Kase, Yuka Suimon, Kan Ishijima, Susumu Ishida

**Affiliations:** Department of Ophthalmology, Faculty of Medicine and Graduate School of Medicine, Hokkaido University, N-15, W-7, Kita-ku, Sapporo, 060-8638 Japan

**Keywords:** Rhegmatogenous retinal detachment, Choroidal melanoma, Choroidal melanomachoroidal detachment, Imaging

## Abstract

**Background:**

Ophthalmologists and retina specialists may consider choroidal detachment if patients with rhegmatogenous retinal detachment present with choroidal elevation. That misdiagnosis may lead to inappropriate treatments, development of tumor cell dissemination, and eventual promotion of patient death. We report a case of a patient with rhegmatogenous retinal detachment associated with choroidal melanoma simulating choroidal detachment according to fundus findings.

**Case presentation:**

A 78-year-old Japanese woman with blurred vision in her right eye was referred to our hospital because of rhegmatogenous retinal detachment with complicated atypical choroidal detachment. Her intraocular pressure was normal with clear anterior chamber. Retinal detachment involving the inferior and nasal retina was observed, and a retinal hole was noted in the same quadrant. A small yellowish choroidal elevation was located in the inferonasal site. Gadolinium-enhanced magnetic resonance imaging revealed enhancement corresponding to the elevation, leading to the identification of a choroidal tumor. Enucleation of the patient’s right eye was eventually performed. The enucleated eye histologically demonstrated malignant melanoma.

**Conclusions:**

If hypotony or an inflammatory sign is absent, ophthalmologists should pay attention to the differential diagnosis of choroidal elevations observed in such patients.

## Introduction

Coincidence of rhegmatogenous retinal detachment (RRD) with choroidal melanoma (CM) is a rare phenomenon, accounting for less than 1% of total CM cases [1, 2]. The phenomenon is commonly found in patients around 50–60 years of age; patients younger than 50 might rarely present with that lesion [2]. Causative retinal breaks can be located in the same or different quadrants with choroidal masses [2, 3]. Treatments for CM complicating RRD consist of enucleation or vitreoretinal surgeries following radiotherapy [3]. However, there is a theoretical but unknown risk if the vitreous cavity is entered; hence, caution is required to make the correct diagnosis.

Diagnosis with CM in RRD may be challenging in selected cases. A variety of publications regarding their rare coincidence have shown choroidal tumors to be relatively easy to identify, which were classified as medium- or large-sized tumors [2, 3]. Unless the size of tumors is medium/large, clinical diagnosis of the choroidal elevation might be confused as choroidal detachment (CD) in patients with RRD. In fact, it is likely for vitrectomy to be conducted as an initial treatment for such patients with RRD who were diagnosed with RRD coincident with CM later [4]. We report a case of a patient with RRD associated with CM, simulating CD according to fundus findings.

## Case presentation

**Fig. 1:**
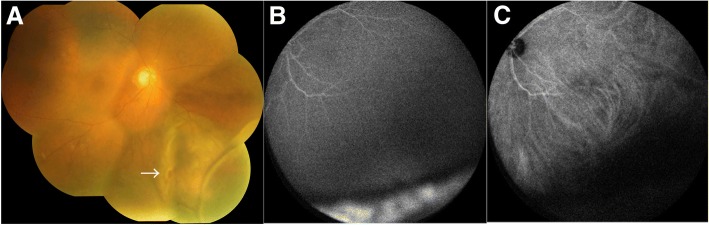
Fundus (**a**), fluorescein angiography (FA) (**b**), and indocyanine green angiography (IA) (**c**) at a late phase at the initial presentation. Retinal detachment involving the inferior and nasal retina is present, where a retinal hole is observed (*arrow* in **a**). FA demonstrates hyperfluorescence in the inferior and nasal retina corresponding to the retinal detachment (**b**), where IA reveals hypofluorescence (**c**)

**Fig. 2:**
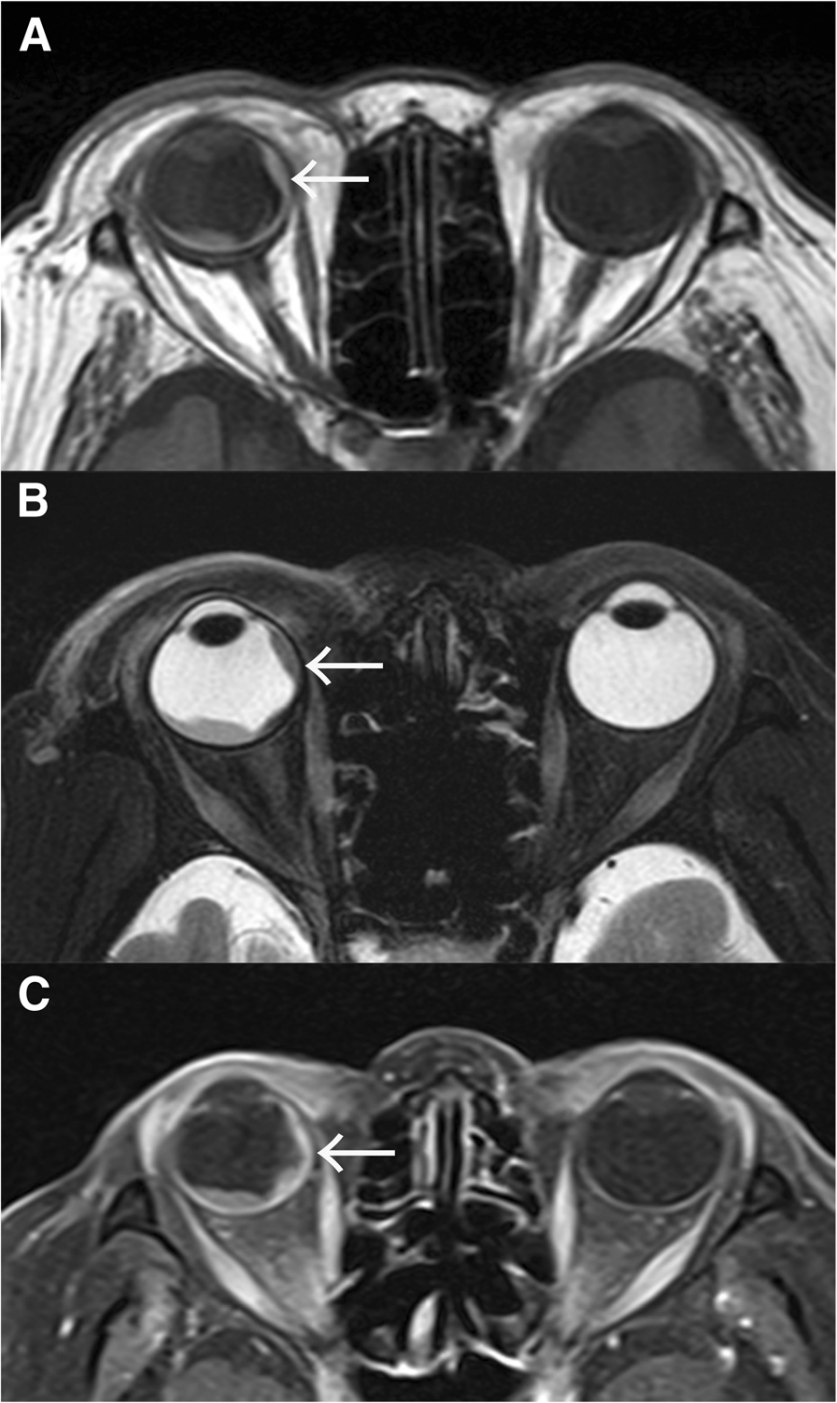
T1-weighted (**a**), T2-weighted (**b**), and gadolinium-enhanced (**c**) magnetic resonance images (MRIs) obtained before enucleation. **a** T1-weighted MRI displays relatively high intensity at the site of the tumor (*arrow*), whereas retinal detachment reveals isointensity in the inferior retina. **b** T2-weighted MRI displays low intensity in the nasal retina where the choroidal tumor situates (*arrow*). **c** The tumor is enhanced by gadolinium (arrow)

A 78-year-old Japanese woman who complained of blurred vision in her right eye visited an ophthalmology clinic. Because RRD was suspected, she was referred to a nearby vitreoretinal surgery center. When RRD with choroidal elevation was noted in the center, the ophthalmologist evaluated CD as atypical. She was eventually referred to our hospital in June 2015. Her medical history revealed nothing of note. Ophthalmological findings demonstrated visual acuity of 4/20 in the right eye (OD) and 10/20 in the left eye (OS). The patient’s intraocular pressure was 12 mmHg OD and 16 mmHg OS. Slit-lamp examination demonstrated clear anterior chambers and senile cortical cataracts in both eyes. Mild pigmented anterior vitreous cells were noted OD. Fundus examination displayed retinal detachment involving the inferior and nasal retina, where a round retinal hole was noted OD (Fig. 1a, arrow). A yellowish flat choroidal elevation was located nasally from the retinal hole. Optical coherence tomography displayed mild subretinal fluid in the macula OD. Fluorescein angiography (FA) revealed hyperfluorescence corresponding to retinal detachment without nonperfusion areas in the fundus (Fig. 1b). Indocyanine green angiography (IA) demonstrated hypofluorescence in the nasal quadrant corresponding to the location of tumor and/or RRD (Fig. 1c). B-mode echography revealed mild choroidal elevation OD with the height of the tumor less than 2 mm. Magnetic resonance imaging (MRI) displayed marginally high and low intensities with T1 and T2 weighting in the choroidal tumor, whereas relatively low intensities and isointensities were detected in the retinal detachment compared with those in the tumor, respectively (Fig. 2a, b; arrows). The choroidal tumor was enhanced by gadolinium (Gd) (Fig. 2c, arrow).

The patient was clinically diagnosed with CM complicating RRD. Systemic imaging modalities did not show abnormalities, except in her right eye. She underwent linear accelerator radiation therapy by X-ray (total dosage, 70 Gy) in a nearby hospital because she refused enucleation and other possible treatments. However, because she had severe ocular pain due to a persistent corneal ulcer, she eventually underwent enucleation in October 2015. Histological findings demonstrated typical mixed-type malignant melanoma with a low Ki-67-positive rate. However, liver metastasis developed about 1½ years after the enucleation. The patient was fine without systemic treatments based on her will in December 2017, and she is being observed in a nearby palliative care center.

## Discussion

CD, which is involved with inflammatory pathogenesis [5], manifests as choroidal elevations in the fundus of patients with RRD. Although the frequency of RRD associated with CD differs with patients’ race, ranging from less than 5% to 20% [5], the two situations are more commonly seen than CM with RRD. Therefore, vitreoretinal surgeons should consider CD first when choroidal elevations are noted in patients with RRD. Misdiagnosis of CD in RRD with CM should therefore be avoided in terms of different treatment strategies and dissemination of malignant tumor cells. In fact, our patient was initially diagnosed with RRD associated with CD located in the same quadrant, but fortunately any treatment did not begin until various imaging examinations were completed.

Clinical features in CM and CD coincident with RRD include their different frequency. Moreover, RRD associated with CD reveals hypotony with inflammatory signs such as increased flare levels in the anterior chamber and vitreous opacity in most cases. Hypotony is a useful finding to consider in the differential diagnosis; however, the findings of increased flare levels in the anterior chamber are not helpful if the tumor volume/size are medium or large in CM [6]. Furthermore, the presence of anterior vitreous cells is not specific for CD, because CM harbors an inflammatory nature. Thus, FA and IA should be performed to distinguish CM from CD. In our patient, however, the angiographic findings did not provide useful information, because the size of the tumor was small, and it was located at the peripheral retina. In our patient, Gd-enhanced MRI findings were the most reliable basis for making a clinical diagnosis of CM.

## Conclusions

When RRD complicating choroidal elevation without inflammatory signs or hypotony is seen, further testing with imaging modalities such as B-mode echography and Gd-enhanced MRI is needed to exclude CM. Those imaging findings clarify whether malignant solid tumor or CD causes choroidal elevations in patients with RRD.

## References

[1] Wang WJ, Schepens CL, Albert DM. Choroidal melanoma associated with rhegmatogenous retinal detachment. Ophthalmic Surg. 1984;15:302–5. 6728403

[2] Wilson GA, Clemett RS. Simultaneous choroidal melanoma and rhegmatogenous retinal detachment. Clin Exp Ophthalmol. 2001;29:94–6. 10.1046/j.1442-9071.2001.d01-11.x 11341455

[3] Lakosha H, Simpson R, Wong D. Choroidal melanoma and rhegmatogenous retinal detachment. Can J Ophthalmol. 2000;35:151–3. 10.1016/s0008-4182(00)80010-1 10812486

[4] Haimovici R, Mukai S, Schachat AP, Haynie GD, Thomas MA, Meredith TA, Gragoudas ES. Rhegmatogenous retinal detachment in eyes with uveal melanoma. Retina. 1996;16:488–96. 10.1097/00006982-199616060-00004 9002131

[5] Dai Y, Wu Z, Sheng H, Zhang Z, Yu M, Zhang Q. Identification of inflammatory mediators in patients with rhegmatogenous retinal detachment associated with choroidal detachment. Mol Vis. 2015;21:417–27. PMC4443581 26015767

[6] Castella AP, Bercher L, Zografos L, Egger E, Herbort CP. Study of the blood-aqueous barrier in choroidal melanoma. Br J Ophthalmol. 1995;79:354–7. 10.1136/bjo.79.4.354 PMC505101 7742283

